# Molecular apocrine tumours in EORTC 10994/BIG 1-00 phase III study: pathological response after neoadjuvant chemotherapy and clinical outcomes

**DOI:** 10.1038/s41416-019-0420-y

**Published:** 2019-03-22

**Authors:** Hervé Bonnefoi, Gaetan MacGrogan, Coralie Poncet, Richard Iggo, Fanny Pommeret, Thomas Grellety, Denis Larsimont, Véronique Bécette, Olivier Kerdraon, Frédéric Bibeau, Jean-Pierre Ghnassia, Jean-Michel Picquenot, Jeremy Thomas, Jean-Christophe Tille, Leen Slaets, Alexandre Bodmer, Jonas Bergh, David Cameron, and on behalf of the EORTC 10994/BIG 1-00 study investigators

**Affiliations:** 1 Department of Medical Oncology, Institut Bergonié Unicancer, University of Bordeaux, INSERM U1218, INSERM CIC1401, Bordeaux, France; 2 Department of BioPathology, Institut Bergonié Unicancer, INSERM U1218 Bordeaux, France; 3 European Organisation for Research and Treatment of Cancer EORTC) Headquarters, Brussels, Belgium; 4 Institut Bergonié Unicancer, INSERM U1218 Bordeaux, France; 5 Department of Pathology, Institut Jules Bordet, Brussels, Belgium; 6 Department of Pathology, Institut Curie—Hôpital René Huguenin, Saint-Cloud, France; 7 Department of Pathology, Centre René Gauducheau, Institut de Cancérologie de l’Ouest, Nantes, France; 8 Department of Pathology, Institut de Cancérologie de Montpellier (ICM), Montpellier, France; 9 Department of Pathology, Centre Paul Strauss, Strasbourg, France; 10 Department of Pathology, Centre Henri Becquerel, Rouen, France; 11 Department of Pathology, Edinburgh Cancer Centre, University of Edinburgh, Edinburgh, United Kingdom; 12 Department of Pathologie, Hôpitaux Universitaires de Genève (HUG), Geneva, Switzerland; 13 Swiss Group for Clinical Cancer Research (SAKK), Bern, Switzerland; 14 Department of Oncology, Hôpitaux Universitaires de Genève (HUG), Geneva, Switzerland; 15 Swedish Breast Cancer Group (SweBCG), Stockholm, Sweden; 16 Department of Oncology and Pathology, Karolinska Institutet, Stockholm, Sweden; 17 Anglo-Celtic Cooperative Oncology Group (ACCOG), Edinburgh, United Kingdom; 18 Department of Medical Oncology, Edinburgh Cancer Centre, University of Edinburgh, Edinburgh, United Kingdom

**Keywords:** Biological sciences, Breast cancer

## Abstract

**Background:**

We explored, within the EORTC10994 study, the outcomes for patients with molecular apocrine (MA) breast cancer, and defined immunohistochemistry (IHC) as androgen-receptor (AR) positive, oestrogen (ER) and progesterone (PR) negative. We also assessed the concordance between IHC and gene expression arrays (GEA) in the identification of MA cancers.

**Methods:**

Centrally assessed biopsies for AR, ER, PR, HER2 and Ki67 by IHC were classified into six subtypes: MA, triple-negative (TN) basal-like, luminal A, luminal B HER2 negative, luminal B HER2 positive and “other”. The two main objectives were the pCR rates and survival outcomes in the overall MA subtype (and further divided by HER2 status) and the remaining five subtypes.

**Results:**

IHC subtyping was obtained in 846 eligible patients. Ninety-three (11%) tumours were classified as the MA subtype. Both IHC and GEA data were available for 64 patients. In this subset, IHC concordance was 88.3% in identifying MA tumours compared with GEA. Within the MA subtype, pCR was observed in 33.3% of the patients (95% CI: 29.4–43.9) and the 5-year recurrence-free interval was 59.2% (95% CI: 48.2–68.6). Patients with MA and TN basal-like tumours have lower survival outcomes.

**Conclusions:**

Irrespective of their HER2 status, the prognosis for MA tumours remains poor and adjuvant trials evaluating anti-androgens should be considered.

## Background

Several gene expression array (GEA) studies have identified a breast cancer subtype characterised by the expression of the androgen receptor (AR), absence of the oestrogen receptor α (ER) and expression of many genes that are expressed in ER-positive luminal tumours.^1–3^ We named these tumours “molecular apocrine” (MA) as they have an increased androgen signalling expression profile and some, but not all, morphological hallmarks of apocrine tumours.^1^ In approximately two-thirds of the cases, these tumours are human epidermal growth factor receptor 2 (HER2) positive; the importance of this HER2-positive AR-driven group of tumours has been recently highlighted.^4^ The remainder are HER2 negative and are part of the heterogeneous triple-negative breast cancer (TNBC) group.

In both HER2-positive and HER2-negative subgroups of MA tumours, prospective trials evaluating anti-androgens in patients with advanced breast cancer are ongoing. In these trials, MA tumours are identified using an immunohistochemical (IHC) definition. In the HER2-negative subgroup, three prospective clinical trials demonstrated antitumour efficacy with anti-androgen treatment^5–7^ and with long-term responders.^8^ In the HER2-positive subgroup, encouraging preliminary results have been reported from a Simon 2-stage phase-two study.^9^ These data have reinforced the interest in the MA subtype and the logical next step would be to evaluate these anti-androgen treatments in patients with MA early breast cancer, at least in the HER2-negative group.

Before considering adjuvant studies, there is a need to better understand the frequency of the MA subtype and its natural history. Previously published EORTC 10994/BIG 1-00 study^10^ offered an excellent opportunity to explore the outcomes for patients with MA tumours compared with other subtypes using an IHC definition. MA tumours were identified using the following definition: AR-positive and ER-, progesterone receptor (PR)-negatives. Moreover, we categorised MA tumours into two subgroups according to HER2 status. This IHC definition is commonly used to identify the MA subtype in prospective therapeutic clinical trials in advanced breast cancer.^5–7^ Other subtypes were defined in a similar way to the St. Gallen 2011 simplified classification^11^ with the exception of the basal-like subtype, which was by definition, AR negative (quadruple negative) in this study.

The MA subtype was initially identified using GEA.^1–3^ For pragmatic reasons, IHC is used to identify this subtype in prospective therapeutic trials. However, the agreement between these two methods has never to our knowledge been assessed. Thus, we determined in a subset of patients included in this substudy, the concordance of IHC compared with this GEA classification in the identification of MA tumours. We used a biologically based GEA classification of breast cancer recently developed to identify MA tumours.^12^

## Materials and methods

### Study design, eligibility and treatment

This was an unplanned analysis within the EORTC 10994/BIG 1-00 neoadjuvant phase III trial, in which 1856 patients were randomised in a 1:1 ratio between six cycles of fluorouracil, epirubicin, cyclophosphamide and a taxane-based regimen, docetaxel for three cycles followed by epirubicin + docetaxel for three cycles, all administered prior to primary surgery as previously described.^10^ Two frozen biopsies from the primary tumour were mandatory for research purposes. Formalin-fixed paraffin-embedded (FFPE) biopsies were performed for diagnostic purposes. Eligible patients for the EORTC 10994/BIG 1-00 trial were women <71 years with histologically proven invasive breast cancer suitable for neoadjuvant chemotherapy, with any large operable or locally advanced/inflammatory breast cancer. At completion of chemotherapy, locoregional treatment was planned in accordance with the guidelines described in the protocol. Treatment was completed with hormonal therapy according to each centre’s policy. Patients with HER2-positive tumours were allowed to participate in adjuvant clinical trials assessing trastuzumab or to receive this treatment in the adjuvant setting once it became standard practice, but none of them received neoadjuvant trastuzumab. The trial was registered with ClinicalTrials.gov number NCT00017095 and approved by national and/or local ethics committees in all participating centres. Before registration, all patients signed an informed consent for the trial and for mandatory p53 gene assessment on tumour samples. In addition, patients were asked to consent for optional biological research on their tumour samples.

For the substudy that is the subject of this report, a subgroup of the initial population of 1856 patients was selected based on the following criteria: (i) patients eligible for the main EORTC 10994/BIG 1-00 trial; (ii) patients who received at least one cycle of neoadjuvant chemotherapy and who did not receive radiotherapy before surgery; (iii) patients who agreed to consent for optional biological research on their tumour samples and (iv) patients with sufficient tumour in their pretreatment core biopsies and whose tumour subtype was identified based on the central analysis of their biopsies included in the ancillary tissue microarray (TMA) study.

### Histopathological assessment

Histological type and grade were assessed locally by pathologists at each participating centre and the data were collected on case report forms in the context of the EORTC 10994 trial. Pathological response was assessed by local pathologists after completion of the neoadjuvant chemotherapy. No central pathology review was performed either for histological type and grade at diagnosis or pathological response at surgery.

### Construction of TMAs

Breast cancer FFPE core biopsies taken at diagnosis before neoadjuvant chemotherapy were retrospectively collected and sent to Institut Bergonié by different participating centres. All core biopsies were reviewed on H- and E-stained sections, and representative tumour areas were selected for TMA construction. For each case, three 0.6-mm-diameter tumour cores were used. The TMA was constructed using a tissue micro-arrayer (Alphelys France). Evaluation of the entire section was performed by a board-certified pathologist (G.M.G.).

### IHC and dual detection in situ hybridisation methods and interpretation

Tumour phenotype concerning AR, ER, PR and HER2 status, and proliferation status (Ki67) were defined on TMA. AR was scored as positive if ≥10% of tumour cell nuclei showed a positive signal. This is the commonly used cut-off.^5–7^ ER and PR were scored negative if <1% of tumour cells were positive. For Ki67, the results were given by % of positive cells. The threshold used to define high Ki-67 expression was ≥14%.^13^ For HER2, the results were given by % of positive cells and the intensity of staining. The final HER2 status was scored according to the ASCO/CAP recommendations.^14^ An IHC3 + score was considered positive. An IHC2+score was considered equivocal. It was then retested by silver in situ hybridisation. Cases with ≥6 *HER2* copies per cell nucleus were considered positive. Details for IHC staining of ER, PR, HER2, Ki67 and AR are provided in Supplementary Table 1.

### Simplified breast cancer molecular subtypes classification

Tumours were classified into six subtypes: MA, triple-negative basal-like (as named in the first GEA classification),^15^ luminal A, luminal B HER2 negative, luminal B HER2 positive and non-luminal non-MA HER2 positive. This classification is detailed in Table 1. The MA subtype was further divided into two subgroups according to HER2 status: positive or negative. The luminal group (ER and/or PR positive, any HER2 status and any Ki67) was further divided in two subgroups according to AR status: positive or negative.

**Table 1:** Simplified breast cancer molecular subtypes classifications (including the molecular apocrine subtype)

Classification in six IHC subtypes	Classification in three IHC subtypes for the comparison to GEA	AR(1)	ER/PR(2)	HER2(3)	Ki67(4)
MA	MA	Positive	Both negative	Any	Any
Luminal A	Luminal	Any	ER and/or PR positive	Negative	Low
Luminal B HER2 negative		Any	ER and/or PR positive	Negative	High
Luminal B HER2 positive		Any	ER and/or PR positive	Positive	Any
Triple-negative basal-like	Basal-like	Negative	Both negative	Negative	Any
Non-luminal and non-MA HER2 positive		Negative	Both negative	Positive	Any

*IHC* immunohistochemical, *GEA* gene expression array, *AR* androgen receptor, *ER* oestrogen receptor, *PR* progesterone receptor, *HER2* human epidermal growth receptor 2, *MA* molecular apocrine-like subtype

(1) AR positive ≥ 10%

(2) ER and PR negative < 1%

(3) HER2 positive: immunohistochemistry (IHC) 3 + or IHC2 + and dual detection in situ hybridisation (DDISH)

(4) Ki67 high ≥ 14%

### TP53 status

TP53 status from frozen biopsies was assessed using a yeast functional test as previously described.^16,17^

### GEA analysis

Microarray data from our previous studies^1,18^ were downloaded from the NCBI GEO database using accession numbers GSE1561 and GSE6861. A biology-based classification of breast cancer was developed using a mammary lineage model (Supplementary Figure 1).^12^ The first step in the classification splits tumours into hormone-sensing tumours (ten transcripts, including ESR1, AR and FOXA1) and secretory cell tumours (nine transcripts, including ELF5, FOXC1 and KLF5). The second step splits hormone-sensing tumours into classic ER+luminal tumours and MA tumours. To separate luminal from MA tumours, 30 genes were selected based on correlation with ESR1 expression, half of them showing positive (luminal) and the other half showing a negative (MA) correlation. Using these 30 genes, luminal and apocrine scores were created. This classification uses a total of 49 preselected transcripts (Supplementary Figure 1). Of note, ERBB2 is not in the list. The tumours were assigned to luminal, MA and basal (LAB) classes as described.^12^ The LAB classification includes a fourth category, “unknown” or “non-interpretable”, for tumours that are too close to the thresholds separating classes to be assigned any particular class confidently. Full details of this classification can be found in the publication.^12^

### Objectives and end-point definitions

The two main objectives were to describe the pathological complete response (pCR) rates and to report the survival outcome measures, recurrence-free interval (RFI), distant RFI (DRFI) and overall survival (OS): (i) in the MA subtype (in the overall MA population and in the two subgroups according to HER2 status); (ii) in the remaining five subtypes and (iii) within the luminal group (three subtypes: luminal A, luminal B HER2 negative and luminal B HER2 positive), in the two subgroups according to AR status (any HER2 and Ki67 status). As an additional aim, we also assessed in a subset of patients the agreement of IHC to identify MA cancers compared with the gold standard GEA.

pCR was defined as no evidence of residual invasive cancer (or very few scattered tumour cells left) with or without residual ductal carcinoma in situ and negative axillary lymph nodes (ypT0/is ypN0). Patients whose tumour progressed on neoadjuvant chemotherapy and patients who did not undergo surgery or with missing information on the surgical pathology report were considered as having “no pCR”.

The survival endpoints were defined according to the standardised definitions for efficacy end-point system.^19^ RFI was measured as time from randomisation to progression on chemotherapy, ipsilateral invasive breast (local) recurrence, regional recurrence (chest wall and regional nodes: axillary, internal mammary, infraclavicular and supraclavicular nodes), distant recurrence or death due to breast cancer and/or treatment toxicity, whichever came first. DRFI was calculated as the time from randomisation to distant recurrence or death due to breast cancer and/or treatment toxicity, whichever came first. OS was calculated as the time from randomisation to death due to any cause. In the EORTC 10994/BIG 1-00 trial, both the first locoregional recurrence and the first distant metastasis were registered. Events diagnosed within 2 months were considered as simultaneous, and we chose to declare the site of the first event as the one with the worst prognosis. Patients who did not present with any of the events mentioned above during their follow-up were censored at the time of their last follow-up. Contralateral breast cancer and the second primary invasive cancer (non-breast) were not considered as primary events.

### Statistical analysis

A statistical analysis plan was prospectively defined. All the statistical analyses were performed using SAS version 9.4 (SAS Institute Inc., Cary, NC, USA).

### pCR analysis

A logistic regression model was used to estimate the effect of a subtype on the odds of having a pCR. The associated exact 95% Clopper–Pearson confidence interval and *p* value based on the Wald method were also presented. Three logistic models were conducted: (i) comparing the six simplified subtypes using the luminal A as the reference group, (ii) within the MA subtype, comparing HER2-positive to HER2-negative subgroups and (iii) within the luminal group (three subtypes as mentioned before), comparing AR-positive to AR-negative subgroups.

### Survival outcomes

Time-to-event endpoints were analysed per Kaplan–Meier method reporting a 5-year estimate and the corresponding Kaplan–Meier curve. *p* Values were based on the log-rank test. Hazard ratios were estimated from a Cox proportional hazard model and the corresponding 95% confidence intervals (CI) (Wald method) were added.

### Concordance between IHC and GEA subtype classification

In a subset of patients with both subtype classifications based on IHC or GEA methods, the proportion of concordant subtype classification and Kappa agreement coefficient, as well as their 95% confidence intervals were estimated.

## Results

Of the 1856 patients originally randomised, core biopsies of 1092 eligible patients were centralised in Bordeaux and available for the TMA construct. A total of 846 patients with a tumour classified in one of the six IHC-based subtypes (Table 1) based on this TMA were included in this substudy. The reasons for ineligibility are shown in the Consort diagram (Supplementary Figure 2). Baseline characteristics and treatment of patients included in this analysis (eligible) and those excluded are presented in Supplementary Table 2 (significant *p* values below 0.05 are indicated). The median follow-up of the patients included in this substudy was 56 months from the date of randomisation. We will first describe the MA population.

### MA tumours

A total of 93/846 (11.0%) eligible tumours were classified in the MA subtype. Baseline characteristics and treatment are reported in Supplementary Table 4. Median age was 54.1 years. Ki67 was high in 81.5% of the patients (75/92) and TP53 status was mutated in 72.1% (49/68) patients. Approximately one-third of MA tumours were HER2 negative (32/92) and two-thirds were HER2 positive (59/92) and one case was equivocal. MA HER2-negative tumours represented 25.4% of all triple-negative breast cancers (32/126). Patient and tumour characteristics were compared between HER2-positive and HER2-negative groups. MA HER2-positive tumours presented more frequently with a high Ki67. All the other characteristics were similar between the two groups except nodal status and Ki67.

A pCR was observed in 31 of 93 (33.3% [95% CI: 23.9–43.9]) patients with MA tumours (Table 2). pCR rates were not significantly different between HER2-negative and HER2-positive subgroups (odds ratio HER2 positive versus HER2 negative 1.31 ([95% CI: 0.51–3.36]; *p* = 0.57)).

**Table 2:** pCR rates by simplified breast cancer molecular subtype

	*Patients (N* *=* *846)*	No pCR (%)	No data on residual tumour^a^ (%)	pCR (%) [95% CI]	Odds ratio (95% CI)
MA					
Any HER2 status^b^	93	58 (62.4)	4 (4.3)	31 (33.3) [23.9–43.9]	5.26 (2.78–9.96)
HER2 negative	32	21 (65.6)	2 (6.3)	9 (28.1)	
HER2 positive	59	37 (62.7)	2 (3.5)	20 (33.9)	
Triple-negative basal-like	94	55 (58.5)	7 (7.4)	32 (34.0) [24.6–44.5]	5.43 (2.88–10.25)
Luminal A	219	199 (90.9)	1 (0.5)	19 (8.4) [5.3–13.2]	1.00
Luminal B HER2 negative	323	279 (86.4)	3 (0.9)	41 (12.7) [9.2–16.8]	1.53 (0.86–2.72)
Luminal B HER2 positive	110	77 (70.0)	3 (2.7)	30 (27.3) [19.2–36.6]	3.95 (2.10–7.41)
Non-luminal and non-MA HER2 positive	7	4 (57.1)	0 (0.0)	3 (42.9) [10.0–81.6]	7.89 (1.64–37.91)
*p* Value^c^					<0.001

^a^No surgery performed (progression on neoadjuvant chemotherapy), considered as no pCR in the logistic regression model

^b^Two patients with equivocal or missing HER2, not included in the subgroups by HER2 status

^c^*p* Value for Wald test of a difference between the six subtypes using a logistic regression model

*MA* molecular apocrine, *pCR* pathological complete response, *CI* confidence interval, *HER2* human epidermal growth factor receptor 2

**Fig. 1:**
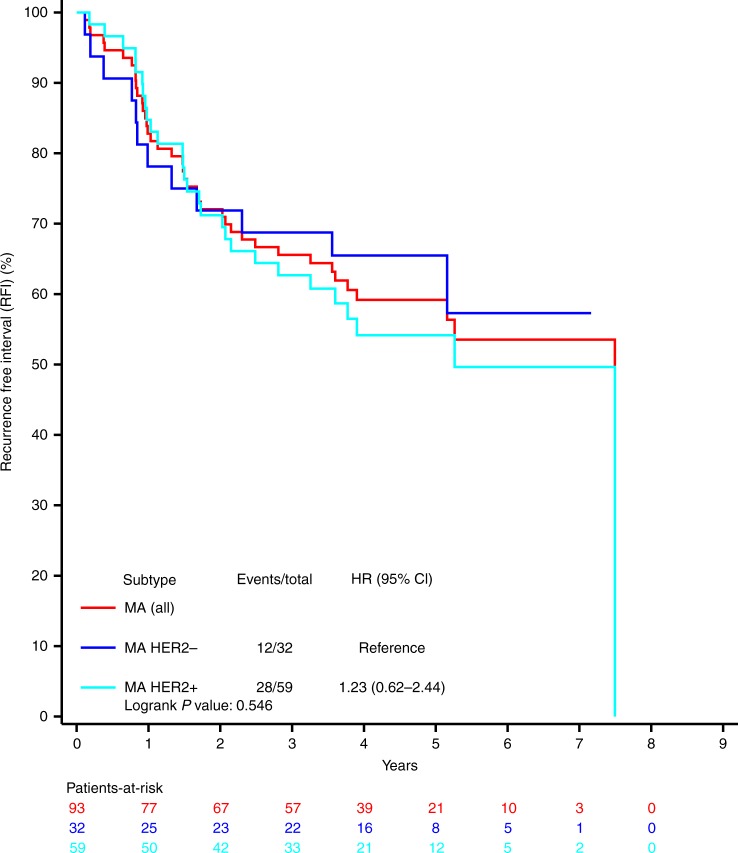
Recurrence-free interval in the molecular apocrine subtype (any HER2 status, HER2-positive and HER2-negative subgroups). HR hazard ratio, CI confidence interval, MA molecular apocrine, HER2− human epidermal growth factor receptor 2 negative, HER2+ human epidermal growth factor receptor 2 positive

The RFI, DRFI and OS curves are shown in Fig. 1 and Supplementary Figures 3 and 4. The 5-year estimate of the RFI rate was 59.2% (95% CI: 48.2–68.6) (Fig. 1 and Supplementary Table 3). Within the MA subtype, survival outcome measures were not statistically different between HER2-positive and HER2-negative subgroups (Fig. 1 and Supplementary Table 5). Approximately, one-third of the first events in MA cancers were locoregional recurrences (Supplementary Table 6). Patterns of distant relapses are reported in Supplementary Table 7.

### Other molecular subtypes and comparison with MA subtypes

Baseline characteristics and treatment are reported in Supplementary Table 9. Patients with MA tumours when compared with other molecular subtypes were older and more often postmenopausal.

pCR rates differed significantly (*p* < 0.001) across intrinsic subtypes, with the lowest rate for luminal A (8.7% [95% CI: 5.3–13.2]) and the highest rates for MA and triple-negative basal-like (33.3% [95% CI: 23.9–43.9] and 34.0% [95% CI: 24.6–44.5], respectively) (Table 2). The pCR rate of HER2-positive non-luminal and non-MA tumours was high (42.9% [95% CI: 10.0–81.6]) but the number of patients in this group is very small.

**Fig. 2:**
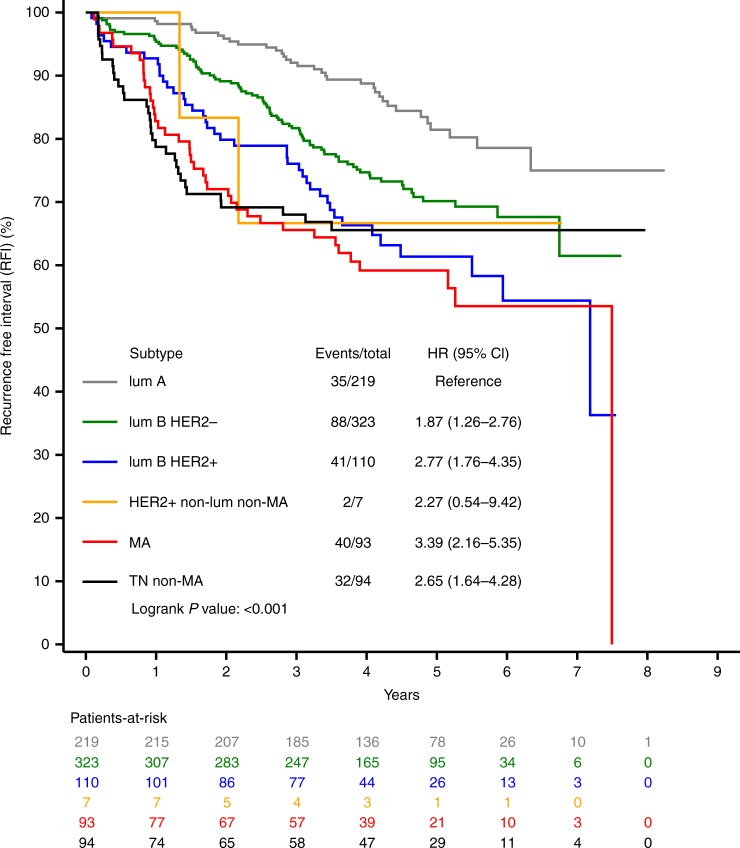
Recurrence-free interval in the six subtypes. HR hazard ratio, CI confidence interval, lum A luminal A, lum B luminal B, HER2– human epidermal growth factor receptor 2 negative, HER2+ human epidermal growth factor receptor 2 positive, MA molecular apocrine, TN triple negative

Patients with MA and triple-negative basal-like tumours showed the lowest 5-year RFI, DRFI and OS estimates (Fig. 2, Supplementary Table 3, Supplementary Figures 5 and 6).

Within luminal subtypes, 8.6% (luminal A) to 18.2% patients (luminal B HER2 negative) experienced locoregional recurrence as the first event contributing to RFI. In non-luminal subtypes, including MA tumours, one-third of the patients experienced locoregional recurrence as the first event contributing to RFI (Supplementary Table 6).

Patterns of distant relapses by simplified breast cancer subtypes are reported in Supplementary Table 7. Compared with patients with luminal tumours, patients with MA tumours presented more often with visceral metastasis (*p* = 0.0343) and less often with bone metastasis (*p* = 0.0006) (Supplementary Table 8).

### Analysis of the ER and/or PR-positive group by AR status

Within the luminal group, 93.7% of the patients (599/639) were AR-positive. pCR rates were not statistically different from AR status (odds ratio 0.62 [95% CI: 0.27–1.39]; *p* = 0.242) (Supplementary Table 10). RFI, DRFI and OS were not statistically different from AR status (Supplementary Table 11).

### Concordance of immunohistochemistry with gene expression to identify MA cancers

We compared the gene expression-based LAB classification^12^ with IHC classification in a subset of 64 patients for whom GEA and IHC data were available. Note that the selection of cases for GEA was not random: it was enriched for ER-negative tumours. By definition, the LAB classification splits tumours into only three groups: luminal, MA and basal-like (Table 1). Hence, to compare it with IHC classification, we assigned cases to three IHC groups (luminal, MA and basal-like) based on IHC for ER, PR and AR. In four patients, the gene expression values were too close to the thresholds for the tumours to be assigned confidently to any particular group, leaving 60 samples for comparison with the IHC data. The concordance was 88.3% (95% CI: 80.2–96.5). The Kappa agreement coefficient between IHC and GEA methods to identify the LAB MA subtype was 0.82 (95%CI: 0.694–0.945) (Table 3).

**Table 3:** Molecular subtypes identified by gene expression array and immunohistochemistry for oestrogen, progesterone and androgen receptors

	GEA classification			
	Luminal (*N* = 25)	MA (*N* = 14)	Basal-like (*N* = 21)	Total (*N* = 60)
	*N* (%)	*N* (%)	*N* (%)	*N* (%)
*IHC classification*				
Luminal (*)	23 (38.3)	2 (3.3)	2 (3.3)	27 (45.0)
MA (**)	1 (1.7)	12 (20.0)	1 (1.7)	14 (23.3)
Basal-like (***)	1 (1.7)	0 (0.0)	18 (30.0)	19 (31.7)

*GEA* gene expression array, *IHC* immunohistochemistry, *MA* molecular apocrine

(*) ER-positive and/or PR positive

(**) AR-positive, ER- and PR-negatives

(***) ER-, PR- and AR-negatives

**Fig. 3:**
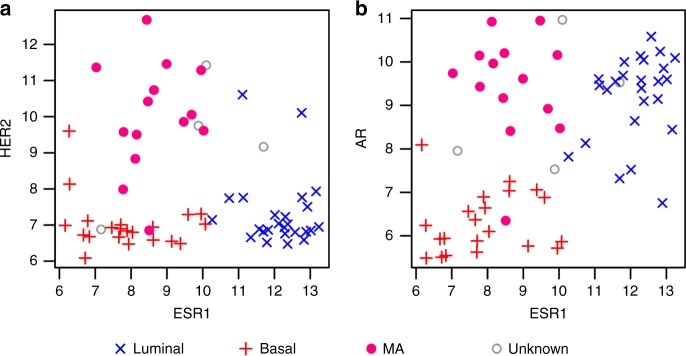
HER2, AR and ESR1 gene expression of individual tumours labelled by LAB class. **a** HER2 and ESR1; **b** AR and ESR1. The points are coloured according to the LAB classification. The gene expression units are arbitrary Affymetrix signal intensities after normalisation with the rma algorithm. HER2 human epidermal growth factor receptor 2, AR androgen receptor, ESR1 oestrogen receptor 1, L luminal, B basal, MA molecular apocrine

HER2 is not used in the LAB classification because it is commonly expressed by both luminal and MA tumours (our hypothesis is that HER2 promotes apocrine metaplasia of luminal cells, leading to a high frequency of HER2 amplification in the MA group). To illustrate the potential limitations of using HER2 to identify MA tumours, we plotted HER2 against ESR1 in Fig. 3a (the tumours are labelled according to the LAB classification in three molecular groups). Tumours expressing high levels of HER2 were indeed classified as MA. but several tumours with high levels of HER2 were luminal (upper right quadrant) or even basal-like, and one MA tumour expressed a low level of HER2. Figure 3b shows the distribution of AR and ESR1 expression in the three molecular groups. The tumours fall into the three expected groups: MA tumours (AR high and ESR1 low) in the upper left quadrant, basal-like tumours (AR low and ESR1 low) in the lower left quadrant and luminal tumours (AR high and ESR1 high) in the upper right quadrant.

Supplementary Figures 7 and 8 highlight the seven discordant cases between the IHC and GEA classifications. Five discordant cases lie close to the thresholds separating the tumour types and can readily be explained by slightly differing the placement of the thresholds by the two approaches. For example, tumours 250 and 337 are classified as MA by gene expression but luminal by IHC; they express AR well but they also express ESR1 at a level almost exactly at the cut-off separating luminal from MA tumours (Supplementary Figure 8). The two remaining discordant cases are outliers (tumours 335 and 856). In these two cases, possible explanations for the discordance are post-transcriptional modifications, tumour heterogeneity or even a sample labelling issue.

In summary, the overall agreement between classification by IHC and gene expression was good, with the disagreements concentrated near the thresholds.

Moreover, we compared LAB classification and a simplified PAM50 classification.^20^ We used 43 out of the 50 genes, which constitute PAM50 successfully mapped to the Affymetrix dataset (seven genes were not present on the U133A chip). We excluded from the comparison tumours which were incomparable (classified as normal by PAM or unknown by LAB), leaving 59 tumours to compare. There was a perfect agreement between the two classifications for the basal tumours. Of note, 89.3% (25/28) of those classified as luminal by PAM were classified as luminal by LAB; three luminal tumours by PAM were MA by LAB. Moreover 78.6% (11/14) of those classified as MA by LAB were classified as HER2-enriched by PAM (Supplementary Table 12).

## Discussion

With a total of 93 MA cancer patients, this is the largest series from a prospective neoadjuvant trial assessing the clinicopathological characteristics, frequency, chemosensitivity and prognosis of this subtype.

In this series, patients diagnosed with MA were older (median age 54.1) and were more often postmenopausal (62.4%) compared with other subtypes. One-third of the first relapses were locoregional. This proportion is similar to that observed in triple-negative basal-like subtype. Two-thirds of MA tumours were HER2 positive and the remainder, HER2 negative. TP53 mutation rate was high (72.1%) and was similar to the one observed in triple-negative basal-like cancers (73.3%).

Regarding frequency, 11% of cancers were classified in the MA subtype. Within the TNBC group, approximately one-quarter were MA (32/126). This information is potentially important when estimating the feasibility of a prospective trial in this molecular subtype in early breast cancer, particularly in the HER2-negative subgroup where no targeted therapy can be offered. In the literature, based on IHC, the frequency of MA tumours in the TNBC group ranges from 21.6% (24/111) in the GBG Gepartrio substudy^21^ to 35.9% (122/339) in the Nurses’ Health study.^22^ Based on GEA, using the TNBCType classification, the frequency of MA tumours is 11.1% (65/587) in a first analysis of 21 publicly available breast cancer GEA datasets performed by the Vanderbilt University group.^23^ In a second Vanderbilt University analysis, the authors simplified their classification from six into four subtypes (TNBCtype-4). Using this refined TNBCtype-4 classification, the frequency is 16% (50/316) in a second analysis performed by this group combining five publicly available GEA datasets of patients treated with neoadjuvant chemotherapy.^24^ Using a different classification algorithm, the frequency of MA is 17.7% (35/198) in a series of tumours collected from U.S. and European sites, with IHC triple-negative status centrally reviewed and GEA analysed in Houston.^25^

The difference in the frequency of AR-positive TNBCs, whether IHC or GEA was used, is difficult to explain, given that there is 88.3% concordance between IHC and GEA to identify the MA subtype in our EORTC series. We believe that this high concordance rate validates the IHC approach taken in this study. Although not perfect, IHC has the advantage of being inexpensive and routinely available in diagnostic histopathology departments. In addition, IHC is commonly used to identify MA cancers in therapeutic trials assessing anti-androgen treatments, from which a significant proportion of patients benefited.^5–7^ We suspect, however, that real progress in the identification of these tumours will come not from analysis of arbitrary signatures or IHC profiles, but rather from a deeper understanding of the underlying biological entity; so we can devise tests that identify that entity on the basis of its essential properties. It was for this reason that we developed the LAB classification.^12^

There are few data reported in the literature regarding the chemosensitivity of MA tumours, in particular, in the HER2-negative subgroup. In the GBG series, the authors used IHC to identify MA tumours. In the TNBC group (*n* = 111), the pCR rates of AR-positive (*n* = 24) and AR-negative (*n* = 87) tumours were similar, 29.2% and 33.3%, respectively.^21^ In our series, within the TNBC group (*n* = 126), the pCR rates of AR-positive (*n* = 32) and AR-negative (*n* = 94) tumours were also similar. In the first Vanderbilt University analysis of TNBC, a total of 42 patients included in two trials received neoadjuvant chemotherapy.^23^ The pCR rates were 14.3% (1/7) and 63.2% (12/19) in MA named luminal AR and basal-like subtypes, respectively.^23^ The MD Anderson Cancer Center (MDACC) group used a similar approach with GEA in a series of 130 evaluable patients with TNBC treated with neoadjuvant chemotherapy.^26^ In luminal AR, basal-like 1 and basal-like 2 subtypes, the pCR rates were 10% (2/20), 52.4% (11/21) and 0% (0/8), respectively. In the second Vanderbilt University analysis, the authors used their simplified TNBCtype-4 classification and assessed the pCR rates in each subtype using data from four publicly available GEA datasets (including the MDACC cohort) corresponding to a total of 306 patients with TNBC.^24^ In this publication, the pCR rate in the luminal AR was 29% (15/52), which is similar to the results observed in the IHC series. It is difficult to explain these apparently different results in pCR rates observed, whether IHC or GEA with TNBCtype or GEA with TNBCtype-4 classifications were used to identify MA tumours. Our interpretation is that, as shown in the LAB classification,^12^ it is easy to separate basal-like from luminal and MA tumours by gene expression, but far more difficult to differentiate luminal and MA tumours. Hence, a possible explanation for the divergent results in pCR rates is that, when using the initial TNBCtype classification tool in the Vanderbilt University and MD Anderson studies,^23,26^ a low-pCR rate in the LAR group was observed because they may have included within this group some classic ER-positive luminal tumours, a subgroup known to have lower pCR rates. By their own admission, the Vanderbilt group acknowledge that they included from 55 to 82% of luminal A or B tumours (identified using the published intrinsic 306-gene set or the PAM50) in the LAR group.^23,24^

In our series, patients with MA and triple-negative basal-like tumours had the worst outcome. Although the distribution of recurrences during the first 3 years was very similar in the two groups, it becomes different after 3 years. In the triple-negative basal-like group (*n* = 94), a plateau was observed but not in the MA group (*n* = 93) (Fig. 2). This plateau is a classic observation in basal-like series. For example, in a French study using GEA, the authors applied their molecular subtype classifier model to a large Affymetrix validation set comprising 2291 breast cancers. On the metastasis-free survival curves, a plateau was observed in the basal-like group (*n* = 264) but not in the MA group (*n* = 146). More than 40% of patients with MA tumours relapsed within 5 years and the survival outcomes were not statistically different between HER2-negative and HER2-positive subgroups. However, a numerical difference was observed for patients with MA HER2-positive tumours. This difference could be explained by the fact that only one-third of patients received adjuvant trastuzumab (EORTC10994/BIG 1-00 accrual period extended from April 2001 to November 2006; Herceptin became standard practice at the end of 2005, which explains why only one-third of patients received this treatment). As far as MA HER2-negative tumours are concerned, the risk of relapse at 5 years in the EORTC study was more than one-third (34.5%). In the two Vanderbilt publications, using GEA-based classifications for TNBCs, the risk of relapse at 5 years of MA cancers was 50% in both series of 62 and 50 MA tumours.^23,24^

Our study has some strengths and limitations. This is the largest series from a prospective trial assessing the frequency and the prognosis of MA tumours, in particular, in TNBCs. The main weakness is that only 45.6% of patients included in the EORTC 10994 study were included in this substudy. In the EORTC study, two frozen biopsies were mandatory, but we did not plan to prospectively collect FFPE blocks prior to neoadjuvant chemotherapy. Hence, these samples were collected retrospectively. However, the characteristics of patients included in this substudy and those who were excluded were similar (Supplementary Table 2). In addition, there was no central assessment of a pathological response. Lastly, we did not use a transcriptomic signature to identify MA tumours and other subtypes. However, as mentioned before, both IHC and GEA methods have a high concordance in the identification of MA tumours (88.3%).

In conclusion, this study demonstrates that the prognosis of MA breast cancers is very poor despite their acceptable rate of pCR after neoadjuvant chemotherapy. Moreover, the MA subtype is frequent, representing approximately 11% of all breast cancers and 25% within the TNBC group. This specific molecular subtype should be considered as an unmet need, particularly in the HER2- negative subgroup, where no targeted therapy can be offered. In the advanced setting, three clinical trials in patients with AR-positive TNBCs have demonstrated an efficacy of anti-androgen treatments.^5–7^ Based on the data reported in these publications. anti-androgen treatments should be evaluated in the adjuvant setting in patients with AR-positive TNBCs.

## Supplementary information

Supplementary material: tables and figures

supplementary table 13

## Data Availability

Data can be accessed through the EORTC data-sharing platform. Data request form is available on the EORTC website: http://www.eortc.org/data-sharing/
