# Supplementary material for: Molecular apocrine tumours in EORTC 10994/BIG 1-00 phase III study: pathological response after neoadjuvant chemotherapy and clinical outcomes
Source: Br J Cancer. 2019 Mar 22;120(9):913–21. doi: 10.1038/s41416-019-0420-y (PMC6734658; doi:10.1038/s41416-019-0420-y)
Supplement: Supplementary file 1 — Supplementary material: tables and figures [file 41416_2019_420_MOESM1_ESM.docx]

| **Supplementary table 1. Immunohistochemical markers used in the study** | | | | | | |
| --- | --- | --- | --- | --- | --- | --- |
| **Marker** | **Clone** | **Vendor** | **Dilution** | **Incubation time (minutes)** | **Pretreatment** | **Staining** |
| Oestrogen Receptor | Sp1 | Ventana | prediluted | 32 | CC1 standard | Ultraview |
| Progesterone Receptor | 1E2 | Ventana | prediluted | 12 | CC1 short | Ultraview |
| Androgen Receptor | Sp107 | Ventana | prediluted | 32 | CC1 standard | Ultraview |
| Her2 | 4B5 | Ventana | prediluted | 12 | CC1 short | Ultraview |
| Ki-67 | 30-9 | Ventana | prediluted | 32 | CC1 standard | Ultraview |

All immunostains were performed on a Benchmark ultra automat (Ventana medical systems). The immunohistochemical techniques for oestrogen receptor (ER), progesterone receptor (PR), androgen receptor (AR), Human epidermal growth factor receptor 2 (Her2) and ki-67 are summarized in supplementary table 1.

*HER2* gene status was determined using a *HER2* SISH technique (INFORM HER2 DNA, Ventana medical systems). *HER2* SISH was performed on an automated instrument, Benchmark Ultra (Ventana medical systems) according to the manufacturer’s protocols for INFORM *HER*2 DNA. The TMA sections were baked at 60°C for 20 minutes. The *HER2* DNA probe was denatured at 95°C for 12 minutes and hybridization was performed at 52°C for 2 hours. After hybridization appropriate stringency washes were performed three times at 72°C. The DNP-labelled *HER2* DNA probe was visualized using a rabbit ant-DNP primary antibody and the UltraView SISH detection kit (Ventana).

Tumours were considered as positive for ER and PR receptors when ≥1 % of cells showed nuclear expression and AR positive when 10% or more tumour cell nuclei were stained. A threshold of 14% of tumour cell nuclear positivity was used to differentiate proliferating (≥14%) from non-proliferating (<14%) tumours (Cheang et al, 2009).

ASCO/CAP recommendations were used to interpret the Her2 immunohistochemical staining (Wolff et al, 2013). *HER2* SISH status was determined by counting the mean number of *HER2* spots per nucleus after analysing 40 tumour cell nuclei par case. A case was considered *HER2* amplified if the mean *HER2* spot count was ≥ 6 and non-amplified if <6. Her2 final status was determined by combining results from the immunohistochemical and the SISH analyses. Tumours were considered Her2 positive when scored 3+ by immunohistochemistry or with six or more HER2 copies per nucleus.

| **Supplementary table 2. Baseline characteristics and treatment of patients included in this analysis (eligible), those who were not included and all patients included in EORTC 10994/BIG 1-00 study** | | | | |
| --- | --- | --- | --- | --- |
|  | **Eligible for analysis** | | **Total (N=1856)** | **p-value** |
|  | **No (N=1010)** | **Yes (N=846)** |  |  |
|  | **N (%)** | **N (%)** | **N (%)** |  |
| **Age** | | | |  |
| <= 40 | 213 (21.1) | 162 (19.1) | 375 (20.2) | 0.5214 |
| 40<-=50 | 380 (37.6) | 335 (39.6) | 715 (38.5) |  |
| 50<-=70 | 417 (41.3) | 349 (41.3) | 766 (41.3) |  |
| Median | 48.6 | 49.0 | 48.8 |  |
| **Menopausal status** | | | |  |
| Premenopausal | 600 (59.4) | 499 (59.0) | 1099 (59.2) | 0.9621 |
| Postmenopausal | 409 (40.5) | 343 (40.5) | 752 (40.5) |  |
| Missing | 1 (0.1) | 4 (0.5) | 5 (0.3) |  |
| **Clinical tumour status** | | | |  |
| T1 | 11 (1.1) | 2 (0.2) | 13 (0.7) | <.0001 |
| T2 | 452 (44.8) | 465 (55.0) | 917 (49.4) |  |
| T3 | 325 (32.2) | 248 (29.3) | 573 (30.9) |  |
| T4 | 222 (22.0) | 130 (15.4) | 352 (19.0) |  |
| Tx | 0 (0.0) | 1 (0.1) | 1 (0.1) |  |
| **Clinical nodal status** | | | |  |
| N0 | 399 (39.5) | 393 (46.5) | 792 (42.7) | 0.0001 |
| N1 | 511 (50.6) | 413 (48.8) | 924 (49.8) |  |
| N2 | 81 (8.0) | 35 (4.1) | 116 (6.3) |  |
| N3 | 15 (1.5) | 5 (0.6) | 20 (1.1) |  |
| Missing | 4 (0.4) | 0 (0.0) | 4 (0.2) |  |
| **Pathological CR (no residual disease in LN)** | | | |  |
| No pCR | 822 (81.4) | 672 (79.4) | 1494 (80.5) | 0.0366 |
| pCR | 152 (15.0) | 156 (18.4) | 308 (16.6) |  |
| No data on residual tumour | 36 (3.6) | 18 (2.1) | 54 (2.9) |  |
| **Histological type** | | | |  |
| Ductal | 831 (82.3) | 715 (84.5) | 1546 (83.3) | 0.4105 |
| Lobular | 110 (10.9) | 90 (10.6) | 200 (10.8) |  |
| Other | 57 (5.6) | 37 (4.4) | 94 (5.1) |  |
| Missing | 12 (1.2) | 4 (0.5) | 16 (0.9) |  |
| **Histological grade** | | | |  |
| Grade 1 | 61 (6.0) | 60 (7.1) | 121 (6.5) | 0.2345 |
| Grade 2 | 430 (42.6) | 429 (50.7) | 859 (46.3) |  |
| Grade 3 | 335 (33.2) | 280 (33.1) | 615 (33.1) |  |
| Missing | 184 (18.2) | 77 (9.1) | 261 (14.1) |  |
| **p53 status** | | | |  |
| Missing | 250 (24.8) | 137 (16.2) | 387 (20.9) | <.0001 |
| Wild Type | 400 (39.6) | 425 (50.2) | 825 (44.5) |  |
| Mutated | 360 (35.6) | 284 (33.6) | 644 (34.7) |  |
| **Type of surgery** | | | |  |
| lumpectomy\quadrantectomy | 368 (36.4) | 396 (46.8) | 764 (41.2) | <.0001 |
| mastectomy | 610 (60.4) | 435 (51.4) | 1045 (56.3) |  |
| Missing | 32 (3.2) | 15 (1.8) | 47 (2.5) |  |
| **Randomized Treatment** | | | |  |
| FEC | 502 (49.7) | 426 (50.4) | 928 (50.0) | 0.8157 |
| T-ET | 508 (50.3) | 420 (49.6) | 928 (50.0) |  |
| **Adjuvant hormonal therapy** | | | |  |
| No | 332 (32.9) | 222 (26.2) | 554 (29.8) | 0.0005 |
| Yes | 640 (63.4) | 614 (72.6) | 1254 (67.6) |  |
| Missing | 38 (3.8) | 10 (1.2) | 48 (2.6) |  |
| **Adjuvant anti-HER2 therapy** | | | |  |
| No | 620 (61.4) | 514 (60.8) | 1134 (61.1) | 0.1162 |
| Yes | 62 (6.1) | 69 (8.2) | 131 (7.1) |  |
| Missing | 328 (32.5) | 263 (31.1) | 591 (31.8) |  |
| p-values from exact Pearson Chi square test; Nominal p-values not adjusted for multiplicity, hypothesis generating only | | | | |

| **Supplementary table 3. Outcomes by simplified breast cancer molecular subtype** | | | | | | | | | | | | | |  |
| --- | --- | --- | --- | --- | --- | --- | --- | --- | --- | --- | --- | --- | --- | --- |
|  | **Recurrence-Free Interval** | | | | **Distant Recurrence-Free Interval** | | | | | **Overall Survival** | | | |  |
|  | **Patients (N)** | **Observed Events (O)** | **% at 5 Years (95% CI)^a^** | **Hazard Ratio (95% CI)^b^** | | **Observed Events (O)** | **% at 5 Years (95% CI) ^a^** | **Hazard Ratio (95% CI) ^b^** | **Observed Events (O)** | | **% at 5 Years (95% CI) ^a^** | **Hazard Ratio (95% CI) ^b^** |  |  |
| MA | 93 | 40 | 59.2 (48.2, 68.6) | 3.39 (2.16, 5.35) | | 33 | 65.7 (54.9, 74.6) | 2.81 (1.73, 4.55) | 19 | | 78.4 (68.1, 85.8) | 2.81 (1.47, 5.35) |  |  |
| Triple negative basal-like | 94 | 32 | 65.6 (54.9, 74.3) | 2.65 (1.64, 4.28) | | 26 | 71.6 (61.1, 79.7) | 2.13 (1.28, 3.57) | 21 | | 76.0 (65.4, 83.8) | 3.15 (1.68, 5.92) |  | |
| Luminal A | 219 | 35 | 81.4 (74.3, 86.7) | 1.00 | | 33 | 83.0 (76.1, 88.1) | 1.00 | 18 | | 94.4 (89.6, 97.0) | 1.00 |  |  |
| Luminal B HER2-negative | 323 | 88 | 70.2 (64.2, 75.3) | 1.87 (1.26, 2.76) | | 80 | 72.2 (66.1, 77.3) | 1.78 (1.18, 2.67) | 44 | | 86.2 (81.0, 90.0) | 1.77 (1.02, 3.07) |  |  |
| Luminal B HER2-positive | 110 | 41 | 61.4 (50.5, 70.6) | 2.77 (1.76, 4.35) | | 32 | 70.1 (59.5, 78.4) | 2.20 (1.35, 3.57) | 15 | | 85.4 (75.1, 91.7) | 1.81 (0.91, 3.59) |  |  |
| Non-luminal and non-MA HER2-positive | 7 | 2 | 66.7 (19.5, 90.4) | 2.27 (0.54, 9.42) | | 1 | 83.3 (27.3, 97.5) | 0.97 (0.13, 7.14) | 1 | | 85.7 (33.4, 97.9) | 1.77 (0.24, 13.26) |  | |
| P-value^c^ | p<0.001 | | | | | p<0.001 | | | p<0.005 | | | |  |  |
| Legend:  ^a^ Non parametric Kaplan-Meier estimate  ^b^ Hazard ratio estimated using a Cox model ; 95%CI using Wald method  ^c^ *P* value from log-rank test  Abbreviations:  MA: molecular apocrine; CI: confidence interval; HER2: human epidermal growth factor receptor 2. | | | | | | | | | | | | |  |  |

| **Supplementary table 4. Baseline characteristics and treatment of molecular apocrine-like tumours** | | | | |  |
| --- | --- | --- | --- | --- | --- |
|  | **Total (N=93)** | **MA HER2- (N=32)** | **MA HER2+ (N=59)** | **HER2 equivocal or NA (N=2)** | **p-value** |
|  | **N (%)** | **N (%)** | **N (%)** | **N (%)** |  |
| **Age** | | | | |  |
| <= 40 | 13 (14.0) | 4 (12.5) | 9 (15.3) | 0 (0.0) | 0.8578 |
| 40<-=50 | 22 (23.7) | 7 (21.9) | 15 (25.4) | 0 (0.0) |  |
| 50<-=70 | 58 (62.4) | 21 (65.6) | 35 (59.3) | 2 (100.0) |  |
| median | 54.1 | 54.9 | 52.3 | 60.1 |  |
| **Menopausal status** | | | | |  |
| Premenopausal | 35 (37.6) | 8 (25.0) | 26 (44.1) | 1 (50.0) | 0.1114 |
| Postmenopausal | 58 (62.4) | 24 (75.0) | 33 (55.9) | 1 (50.0) |  |
| **T stage** | | | | |  |
| T1 | 1 (1.1) | 0 (0.0) | 1 (1.7) | 0 (0.0) | 0.8695 |
| T2 | 50 (53.8) | 18 (56.3) | 32 (54.2) | 0 (0.0) |  |
| T3 | 21 (22.6) | 8 (25.0) | 12 (20.3) | 1 (50.0) |  |
| T4 | 21 (22.6) | 6 (18.8) | 14 (23.7) | 1 (50.0) |  |
| **N stage** |  |  |  |  |  |
| N0 | 35 (37.6) | 13 (40.6) | 21 (35.6) | 1 (50.0) | 0.0446 |
| N1 | 49 (52.7) | 17 (53.1) | 31 (52.5) | 1 (50.0) |  |
| N2 | 7 (7.5) | 0 (0.0) | 7 (11.9) | 0 (0.0) |  |
| N3 | 2 (2.2) | 2 (6.3) | 0 (0.0) | 0 (0.0) |  |
| **Histological type** | | | | |  |
| Ductal | 87 (93.5) | 29 (90.6) | 56 (94.9) | 2 (100.0) | 0.1816 |
| Lobular | 4 (4.3) | 3 (9.4) | 1 (1.7) | 0 (0.0) |  |
| Other | 2 (2.2) | 0 (0.0) | 2 (3.4) | 0 (0.0) |  |
| **Grade** | | | | |  |
| Grade 1 | 2 (2.2) | 2 (6.3) | 0 (0.0) | 0 (0.0) | 0.1899 |
| Grade 2 | 35 (37.6) | 12 (37.5) | 23 (39.0) | 0 (0.0) |  |
| Grade 3 | 45 (48.4) | 14 (43.8) | 29 (49.2) | 2 (100.0) |  |
| Not known or NA | 11 (11.8) | 4 (12.5) | 7 (11.9) | 0 (0.0) |  |
| **KI67** | | | | |  |
| 0% - <14% | 17 (18.3) | 8 (25.0) | 9 (15.3) | 0 (0.0) | 0.009 |
| 14% - 30% | 42 (45.2) | 8 (25.0) | 34 (57.6) | 0 (0.0) |  |
| >30% - 100% | 33 (35.5) | 16 (50.0) | 15 (25.4) | 2 (100.0) |  |
| Missing | 1 (1.1) | 0 (0.0) | 1 (1.7) | 0 (0.0) |  |
| Median (range) | 25 (5-80) | 35 (5-80) | 20 (5-80) | 50 (40-60) |  |
| **TP53 status** | | | | |  |
| Not done   or test failure | 25 (26.9) | 8 (25.0) | 17 (28.8) | 0 (0.0) | 0.7380 |
| Wild Type | 19 (20.4) | 6 (18.8) | 13 (22.0) | 0 (0.0) |  |
| Mutated | 49 (52.7) | 18 (56.3) | 29 (49.2) | 2 (100.0) |  |
| **Type of surgery** | | | | |  |
| Lumpectomy/quadr antectomy | 40 (43.0) | 17 (53.1) | 23 (39.0) | 0 (0.0) | 0.1816 |
| Mastectomy | 50 (53.8) | 13 (40.6) | 35 (59.3) | 2 (100.0) |  |
| No surgery | 3 (3.2) | 2 (6.3) | 1 (1.7) | 0 (0.0) |  |
| **Randomized treatment** | | | | |  |
| FEC | 43 (46.2) | 17 (53.1) | 26 (44.1) | 0 (0.0) | 0.5104 |
| T-ET | 50 (53.8) | 15 (46.9) | 33 (55.9) | 2 (100.0) |  |
| **Adjuvant hormonal therapy** | | | | |  |
| No | 80 (86.0) | 26 (81.3) | 52 (88.1) | 2 (100.0) | 0.7295 |
| Yes | 10 (10.8) | 4 (12.5) | 6 (10.2) | 0 (0.0) |  |
| Missing | 3 (3.2) | 2 (6.3) | 1 (1.7) | 0 (0.0) |  |
| **Adjuvant trastuzumab** | | | | |  |
| No | 59 (63.4) | 17 (53.1) | 40 (67.8) | 2 (100.0) | 0.1289 |
| Yes | 19 (20.4) | 2 (6.3) | 17 (28.8) | 0 (0.0) |  |
| Missing | 15 (16.1) | 13 (40.6) | 2 (3.4) | 0 (0.0) |  |
| Abbreviations:  MA: Molecular apocrine-like; NA: not assessable; HER2: human epidermal growth receptor 2.  p-values from exact Pearson Chi square test; Nominal p-values not adjusted for multiplicity, hypothesis generating only | | | | |  |

| **Supplementary table 5. Survival outcomes of patients with molecular apocrine tumours by HER2 status** | | | | | | | | | | |
| --- | --- | --- | --- | --- | --- | --- | --- | --- | --- | --- |
|  |  | **RFI**  **Recurrence-Free Interval** | | | **DRFI**  **Distant Recurrence-Free Interval** | | | **OS**  **Overall Survival** | | |
| (within MA subtype) | **Patients (N)** | **Observed Events (O)** | **% at 5 Years (95% CI)^a^** | **Hazard Ratio (95% CI)^b^** | **Observed Events (O)** | **% at 5 Years (95% CI)^a^** | **Hazard Ratio (95% CI)^b^** | **Observed Events (O)** | **% at 5 Years (95% CI)^a^** | **Hazard Ratio (95% CI)^b^** |
| MA HER2- | 32 | 12 | 65.5 (46.4, 79.2) | 1.00 | 11 | 68.5 (49.3, 81.6) | 1.00 | 9 | 71.9 (52.9, 84.3) | 1.00 |
| MA HER2+ | 59 | 28 | 54.1 (40.1, 66.2) | 1.23 (0.62, 2.44) | 22 | 62.9 (48.8, 74.1) | 1.07 (0.52, 2.22) | 10 | 81.2 (67.5, 89.5) | 0.54(0.22, 1.34) |
| P-value^c^ | 0.546 | | | | 0.847 | | | 0.178 | | |
| Legend:  ^a^ Non parametric Kaplan-Meier estimate  ^b^ Hazard ratio estimated using a Cox model ; 95%CI using Wald method  ^c^ *P* value from log-rank test  Abbreviations:  CI: confidence interval; HER2: human epidermal growth factor receptor 2 | | | | | | | | | | |

| **Supplementary table 6. Types of first events contributing to recurrence-free interval** | | | | | | | |
| --- | --- | --- | --- | --- | --- | --- | --- |
|  | **lum A (N=35)** | **lum B HER2- (N=88)** | **lum B HER2+ (N=41)** | **HER2+ non-lum non-MA (N=2)** | **MA (N=40)** | **TN basal-like (N=32)** | **Total (N=238)** |
|  | **N (%)** | **N (%)** | **N (%)** | **N (%)** | **N (%)** | **N (%)** | **N (%)** |
| Distant recurrence | 32 (91.4) | 72 (81.8) | 30 (73.2) | 1 (50.0) | 27 (67.5) | 19 (59.4) | 181 (76.1) |
| Loco-regional recurrence | 3 (8.6) | 16 (18.2) | 10 (24.4) | 1 (50.0) | 13 (32.5) | 13 (40.6) | 56 (23.5) |
| BC Death | 0 (0.0) | 0 (0.0) | 1 (2.4) | 0 (0.0) | 0 (0.0) | 0 (0.0) | 1 (0.4) |

In the EORTC 10994 trial, both first locoregional recurrence and first distant metastasis were registered. Events diagnosed within two months were considered as simultaneous and we chose to declare the site of first event as the one with the worst prognosis.

| **Supplementary table 7. Patterns of distant relapses(*) by simplified breast cancer molecular subtype** | | | | | | | |
| --- | --- | --- | --- | --- | --- | --- | --- |
|  | **lum A (N=32)** | **lum B HER2- (N=72)** | **lum B HER2+ (N=30)** | **HER2+ non-lum non-MA (N=1)** | **MA (N=27)** | **TN basal-like (N=19)** | **Total (N=181)** |
|  | **N (%)** | **N (%)** | **N (%)** | **N (%)** | **N (%)** | **N (%)** | **N (%)** |
| Soft tissue | 4 (12.5) | 6 (8.3) | 2 (6.7) | 0 (0.0) | 4 (14.8) | 3 (15.8) | 19 (10.5) |
| Visceral | 14 (43.8) | 38 (52.8) | 16 (53.3) | 0 (0.0) | 20 (74.1) | 9 (47.4) | 97 (53.6) |
| Skeletal | 22 (68.8) | 40 (55.6) | 17 (56.7) | 0 (0.0) | 6 (22.2) | 4 (21.1) | 89 (49.2) |
| CNS | 1 (3.1) | 1 (1.4) | 4 (13.3) | 0 (0.0) | 5 (18.5) (**) | 5 (26.3) | 16 (8.8) |
| Other | 1 (3.1) | 3 (4.2) | 0 (0.0) | 0 (0.0) | 0 (0.0) | 1 (5.3) | 5 (2.8) |

(*) One patient can have more than one metastatic site involved.

(**) 2 patients presented with concomitant other visceral metastasis, 1 with a concomitant locoregional relapse and 2 with isolated CNS relapse.

| **Supplementary table 8. Site of first distant metastasis** | | | |
| --- | --- | --- | --- |
|  | **Luminal (N=134)** | **MA (N=27)** | **P value** |
|  | **N (%)** | **N (%)** |  |
| **Visceral** |  |  |  |
| **no** | 65 (48.5) | 7 (25.9) | 0.0343 |
| **yes** | 68 (50.7) | 20 (74.1) |  |
| **Missing** | 1 (0.7) | 0 (0.0) |  |
| **Skeletal** |  |  |  |
| **no** | 54 (40.3) | 21 (77.8) | 0.0006 |
| **yes** | 79 (59.0) | 6 (22.2) |  |
| **Missing** | 1 (0.7) | 0 (0.0) |  |
| p-values from exact Pearson Chi square test | | | |

| **Supplementary table 9. Baseline characteristics and treatment by simplified breast cancer subtype** | | | | | | | |
| --- | --- | --- | --- | --- | --- | --- | --- |
|  | **Lum A (N=219)** | **Lum B HER2- (N=323)** | **Lum B HER2+ (N=110)** | **HER2+ non-lum non-MA (N=7)** | **MA (N=93)** | **TN basal-like (N=94)** | **Total (N=846)** |
|  | **N (%)** | **N (%)** | **N (%)** | **N (%)** | **N (%)** | **N (%)** | **N (%)** |
| **Age** | | | | | | | |
| <= 40 | 31 (14.2) | 68 (21.1) | 23 (20.9) | 0 (0.0) | 13 (14.0) | 27 (28.7) | 162 (19.1) |
| 40<-=50 | 98 (44.7) | 142 (44.0) | 45 (40.9) | 3 (42.9) | 22 (23.7) | 25 (26.6) | 335 (39.6) |
| 50<-=70 | 90 (41.1) | 113 (35.0) | 42 (38.2) | 4 (57.1) | 58 (62.4) | 42 (44.7) | 349 (41.3) |
| Median | 49.0 | 47.9 | 48.4 | 53.6 | 54.1 | 49.6 | 49.0 |
| **Menopausal status** | | | | | | | |
| Premenopausal | 130 (59.4) | 213 (65.9) | 65 (59.1) | 4 (57.1) | 35 (37.6) | 52 (55.3) | 499 (59.0) |
| Postmenopausal | 87 (39.7) | 109 (33.7) | 44 (40.0) | 3 (42.9) | 58 (62.4) | 42 (44.7) | 343 (40.5) |
| Missing | 2 (0.9) | 1 (0.3) | 1 (0.9) | 0 (0.0) | 0 (0.0) | 0 (0.0) | 4 (0.5) |
| **Clinical tumour status** | | | | | | | |
| T1 | 0 (0.0) | 1 (0.3) | 0 (0.0) | 0 (0.0) | 1 (1.1) | 0 (0.0) | 2 (0.2) |
| T2 | 130 (59.4) | 175 (54.2) | 50 (45.5) | 3 (42.9) | 50 (53.8) | 57 (60.6) | 465 (55.0) |
| T3 | 65 (29.7) | 100 (31.0) | 35 (31.8) | 2 (28.6) | 21 (22.6) | 25 (26.6) | 248 (29.3) |
| T4 | 24 (11.0) | 47 (14.6) | 24 (21.8) | 2 (28.6) | 21 (22.6) | 12 (12.8) | 130 (15.4) |
| Tx | 0 (0.0) | 0 (0.0) | 1 (0.9) | 0 (0.0) | 0 (0.0) | 0 (0.0) | 1 (0.1) |
| **Clinical nodal status** | | | | | | | |
| N0 | 135 (61.6) | 137 (42.4) | 48 (43.6) | 1 (14.3) | 35 (37.6) | 37 (39.4) | 393 (46.5) |
| N1 | 83 (37.9) | 171 (52.9) | 57 (51.8) | 6 (85.7) | 49 (52.7) | 47 (50.0) | 413 (48.8) |
| N2 | 1 (0.5) | 14 (4.3) | 5 (4.5) | 0 (0.0) | 7 (7.5) | 8 (8.5) | 35 (4.1) |
| N3 | 0 (0.0) | 1 (0.3) | 0 (0.0) | 0 (0.0) | 2 (2.2) | 2 (2.1) | 5 (0.6) |
| **Histological type** | | | | | | | |
| Ductal | 162 (74.0) | 277 (85.8) | 96 (87.3) | 6 (85.7) | 87 (93.5) | 87 (92.6) | 715 (84.5) |
| Lobular | 48 (21.9) | 32 (9.9) | 4 (3.6) | 0 (0.0) | 4 (4.3) | 2 (2.1) | 90 (10.6) |
| Other | 9 (4.1) | 12 (3.7) | 9 (8.2) | 1 (14.3) | 2 (2.2) | 4 (4.3) | 37 (4.4) |
| Unknown | 0 (0.0) | 2 (0.6) | 1 (0.9) | 0 (0.0) | 0 (0.0) | 1 (1.1) | 4 (0.5) |
| **Histological grade** | | | | | | | |
| Grade 1 | 34 (15.5) | 19 (5.9) | 5 (4.5) | 0 (0.0) | 2 (2.2) | 0 (0.0) | 60 (7.1) |
| Grade 2 | 152 (69.4) | 180 (55.7) | 39 (35.5) | 3 (42.9) | 35 (37.6) | 20 (21.3) | 429 (50.7) |
| Grade 3 | 16 (7.3) | 94 (29.1) | 50 (45.5) | 4 (57.1) | 45 (48.4) | 71 (75.5) | 280 (33.1) |
| Unknown | 17 (7.8) | 30 (9.3) | 16 (14.5) | 0 (0.0) | 11 (11.8) | 3 (3.2) | 77 (9.1) |
| **ER (central – TMA)** | | | | | | | |
| Negative | 2 (0.9) | 7 (2.2) | 16 (14.5) | 7 (100.0) | 93 (100.0) | 94 (100.0) | 219 (25.9) |
| Positive | 217 (99.1) | 314 (97.2) | 94 (85.5) | 0 (0.0) | 0 (0.0) | 0 (0.0) | 625 (73.9) |
| Missing | 0 (0.0) | 2 (0.6) | 0 (0.0) | 0 (0.0) | 0 (0.0) | 0 (0.0) | 2 (0.2) |
| **PR (central – TMA)** | | | | | | | |
| Negative | 39 (17.8) | 48 (14.9) | 26 (23.6) | 7 (100.0) | 93 (100.0) | 94 (100.0) | 307 (36.3) |
| Positive | 179 (81.7) | 275 (85.1) | 83 (75.5) | 0 (0.0) | 0 (0.0) | 0 (0.0) | 537 (63.5) |
| Missing | 1 (0.5) | 0 (0.0) | 1 (0.9) | 0 (0.0) | 0 (0.0) | 0 (0.0) | 2 (0.2) |
| **AR (central – TMA)** | | | | | | | |
| Negative | 9 (4.1) | 27 (8.4) | 4 (3.6) | 7 (100.0) | 0 (0.0) | 94 (100.0) | 141 (16.7) |
| Positive | 204 (93.2) | 290 (89.8) | 105 (95.5) | 0 (0.0) | 93 (100.0) | 0 (0.0) | 692 (81.8) |
| Missing | 6 (2.7) | 6 (1.9) | 1 (0.9) | 0 (0.0) | 0 (0.0) | 0 (0.0) | 13 (1.5) |
| **HER2 (central – TMA)** | | | | | | | |
| Negative | 219 (100.0) | 323 (100.0) | 0 (0.0) | 0 (0.0) | 32 (34.4) | 94 (100.0) | 668 (79.0) |
| Positive | 0 (0.0) | 0 (0.0) | 110 (100.0) | 7 (100.0) | 59 (63.4) | 0 (0.0) | 176 (20.8) |
| Equivocal | 0 (0.0) | 0 (0.0) | 0 (0.0) | 0 (0.0) | 1 (1.1) | 0 (0.0) | 1 (0.1) |
| Missing | 0 (0.0) | 0 (0.0) | 0 (0.0) | 0 (0.0) | 1 (1.1) | 0 (0.0) | 1 (0.1) |
| **KI67 (central – TMA)** | | | | | | | |
| **Low** | 219 (100.0) | 0 (0.0) | 19 (17.3) | 2 (28.6) | 17 (18.3) | 5 (5.3) | 262 (31.0) |
| **High** | 0 (0.0) | 323 (100.0) | 90 (81.8) | 5 (71.4) | 75 (80.6) | 89 (94.7) | 582 (68.8) |
| **Missing** | 0 (0.0) | 0 (0.0) | 1 (0.9) | 0 (0.0) | 1 (1.1) | 0 (0.0) | 2 (0.2) |
| **p53 status** | | | | | | | |
| **Unknow** | 29 (13.2) | 44 (13.6) | 19 (17.3) | 1 (14.3) | 25 (26.9) | 19 (20.2) | 137 (16.2) |
| **Wild Type** | 152 (69.4) | 183 (56.7) | 49 (44.5) | 2 (28.6) | 19 (20.4) | 20 (21.3) | 425 (50.2) |
| **Mutated** | 38 (17.4) | 96 (29.7) | 42 (38.2) | 4 (57.1) | 49 (52.7) | 55 (58.5) | 284 (33.6) |
| **Type of surgery** | | | | | | | |
| **Lumpectomy\quadrantectomy** | 109 (49.8) | 138 (42.7) | 48 (43.6) | 5 (71.4) | 40 (43.0) | 56 (59.6) | 396 (46.8) |
| **Mastectomy** | 109 (49.8) | 182 (56.3) | 59 (53.6) | 2 (28.6) | 50 (53.8) | 33 (35.1) | 435 (51.4) |
| **No surgery** | 1 (0.5) | 3 (0.9) | 3 (2.7) | 0 (0.0) | 3 (3.2) | 5 (5.3) | 15 (1.8) |
| **Randomized treatment** |  |  |  |  |  |  |  |
| **FEC** | 109 (49.8) | 168 (52.0) | 59 (53.6) | 5 (71.4) | 43 (46.2) | 42 (44.7) | 426 (50.4) |
| **T-ET** | 110 (50.2) | 155 (48.0) | 51 (46.4) | 2 (28.6) | 50 (53.8) | 52 (55.3) | 420 (49.6) |
| **Adjuvant hormonal therapy (*)** | | | | | | | |
| **No** | 9 (4.1) | 24 (7.4) | 27 (24.5) | 6 (85.7) | 80 (86.0) | 76 (80.9) | 222 (26.2) |
| **Yes** | 210 (95.9) | 297 (92.0) | 82 (74.5) | 1 (14.3) | 10 (10.8) | 14 (14.9) | 614 (72.6) |
| **Missing** | 0 (0.0) | 2 (0.6) | 1 (0.9) | 0 (0.0) | 3 (3.2) | 4 (4.3) | 10 (1.2) |
| **Adjuvant anti-HER2 therapy** | | | | | | | |
| **No** | 140 (63.9) | 198 (61.3) | 62 (56.4) | 4 (57.1) | 59 (63.4) | 51 (54.3) | 514 (60.8) |
| **Yes** | 1 (0.5) | 4 (1.2) | 42 (38.2) | 3 (42.9) | 19 (20.4) | 0 (0.0) | 69 (8.2) |
| **Missing** | 78 (35.6) | 121 (37.5) | 6 (5.5) | 0 (0.0) | 15 (16.1) | 43 (45.7) | 263 (31.1) |

(*) The decision to prescribe hormonal therapy was taken by investigators based on local IHC assessment. The classification into one of the 6 subtypes was done based on central IHC assessment.

| **Supplementary table 10. pathological complete response rates in the luminal group** ^a^ **by AR status** | | | | | |
| --- | --- | --- | --- | --- | --- |
|  | **Patients (N=639)** ^b^ | **No pCR (%)** | **No data**  **on residual tumour**  **(%)^c^** | **pCR**  **(%)** | **Odds ratio**  **lum AR+ versus lum AR-**  **(95% CI)** |
| Luminal AR+ | 599 | 513 (85.6) | 6 (1.0) | 80 (13.4) | 1.00 |
| Luminal AR- | 40 | 31 (77.5) | 1 (2.5) | 8 (20.0) | 0.62 (0.27, 1.39) |
| P-value^d^ |  | | | | 0.242 |
| Legend:  ^a^ Luminal group includes 3 subtypes: luminal A, luminal B HER2 negative, luminal B HER2 positive  ^b^ 13 patients had luminal tumour with missing AR status and were therefore not considered for these subgroups  ^c^ No surgery performed or missing information on the surgical pathology report; considered as No pCR  ^d^ *P* value for Wald test of a difference between the subtypes using a logistic regression model.  Abbreviations:  pCR: pathological complete response; AR: androgen receptor; CI: confidence interval; HER2: human epidermal growth factor receptor 2. | | | | | |

| **Supplementary table 11. Survival outcomes in the luminal group** ^a^ **by AR status** | | | | | | | | | | |
| --- | --- | --- | --- | --- | --- | --- | --- | --- | --- | --- |
|  | | **Recurrence-Free Interval** | | | **Distant Recurrence-Free Interval** | | | **Overall Survival** | | |
|  | **Patients (N)** | **Observed Events (O)** | **% at 5 Year(s) (95% CI) ^b^** | **Hazard Ratio (95% CI) ^c^** | **Observed Events (O)** | **% at 5 Years (95% CI)^b^** | **Hazard Ratio (95% CI)^c^** | **Observed Events (O)** | **% at 5 Years (95% CI)^b^** | **Hazard Ratio (95% CI)^c^** |
| AR-positive | 599 | 150 | 72.8 (68.5, 76.6) | 1.00 | 133 | 75.5 (71.2, 79.2) | 1.00 | 67 | 89.3 (85.9, 91.9) | 1.00 |
| AR-negative | 40 | 12 | 65.4 (46.0, 79.3) | 1.29 (0.72, 2.32) | 10 | 73.3 (53.4, 85.7) | 1.17 (0.61, 2.23) | 8 | 82.9 (65.1, 92.1) | 1.81 (0.87, 3.77) |
| P-value^d^ | 0.399 | | | | 0.633 | | | 0.107 | | |
| Legend:  ^a^ Luminal group includes 3 subtypes: luminal A, luminal B HER2 negative, luminal B HER2 positive  ^b^ Non parametric Kaplan-Meier estimate  ^c^ Hazard ratio estimated using a Cox model ; 95%CI using Wald method  ^d^*P* value from log-rank test  Abbreviations:  CI: confidence interval; HER2: human epidermal growth factor receptor 2 | | | | | | | | | | |

| Supplementary table 12. Molecular subtypes identified by gene expression array: comparison of LAB and PAM43 classifications | | | | |
| --- | --- | --- | --- | --- |
|  | **GEA classification** | | | |
|  | **Luminal** | **MA** | **Basal** | **Unknown** |
| **PAM classification** |  | | | |
| **LumA** | 15 | 3 | 0 | 1 |
| **LumB** | 10 | 0 | 0 | 0 |
| **Her2-enriched** | 0 | 11 | 0 | 2 |
| **Basal** | 0 | 0 | 20 | 1 |
| **Normal** | 0 | 0 | 1 | 0 |

**Supplementary figure 1. Mammary lineage diagram**

**Supplementary figure 2. CONSORT diagram**

1856 patients included
and randomised(*)

22 patients ineligible for the main EORTC 10994 trial (**)

6 patients who received no neoadjuvant chemotherapy

31 patients received radiotherapy before surgery

738 patients for whom we were unable to centralize FFPE samples

Core biopsies from 1092 eligible patients were centralized in Institut Bergonié, Bordeaux, France

105 patients with insufficient information for central subtyping

141 patients didn’t consent for optional research on sample

846 patients eligible for analysis describing pCR and clinical outcomes

(*) Patients may be ineligible for several reasons

(**) Reasons for ineligibility are detailed in the final analysis of EORTC 10994 trial (Bonnefoi et al Lancet Oncol 2011)

**Supplementary figure 3. Distant recurrence-free interval in the molecular apocrine subtype (any HER2 status, HER2 positive and HER2 negative subgroups)**

**Supplementary figure 4. Overall survival in the molecular apocrine subtype (any HER2 status, HER2 positive and HER2 negative subgroups)**

**Supplementary Figure 5. Distant recurrence-free interval in the six subtypes**

**Supplementary figure 6. Overall survival in the six subtypes**

**Supplementary figure 7. Enlarged view of figure 3B showing AR/ESR1 expression highlighting discordant cases**

Legend:

The points are coloured according to the LAB classification. The labels are placed immediately below the annotated tumours; they contain the EORTC identification number followed by the IHC classification (L, B or MA for luminal, basal-like or molecular apocrine tumours). The gene expression units are arbitrary Affymetrix signal intensities after normalisation with the rma algorithm.

Abbreviations:

AR: androgen receptor; ESR1: estrogen receptor 1; L: luminal; B: basal; MA: molecular apocrine.

**Supplementary figure 8. Distribution of gene expression in the LAB classes:** A, ESR1; B, AR; and C, ERBB2

Legend:

The distributions are normalised to a peak intensity of 1 to simplify visual interpretation of the plots. The gene expression units are arbitrary Affymetrix signal intensities after normalisation with the rma algorithm. The labels (EORTC identification numbers) of the discordant cases are placed on the top of each figure.
